# Penile metastasis from recurrent sarcoma in a teenager: a case report

**DOI:** 10.1186/s12894-019-0511-3

**Published:** 2019-09-02

**Authors:** Chi-Fang Chen, Tzu-Ying Tang, Marcelo Chen, Li-Chen Chen

**Affiliations:** 1 Department of Urology, MacKay Memorial Hospital, No.92, Sec. 2, Zhongshan N. Rd., Zhongshan Dist, Taipei City, 10449 Taiwan; 2 Department of Pathology, Tamsui branch, MacKay Memorial Hospital, No.45, Minsheng Rd., Tamsui Dist, New Taipei City, 25160 Taiwan; 3 School of Medicine, MacKay Medical College, No.46, Sec. 3, Zhongzheng Rd., Sanzhi Dist, New Taipei City, 252 Taiwan

**Keywords:** Secondary penile tumor, Metastasis, Malignant priapism, Sarcoma

## Abstract

**Background:**

Metastatic tumors of the penis are uncommon, and fewer than 500 cases have been reported since 1870. Most penile secondary tumors originate in organs of the genitourinary tract, followed by the gastrointestinal tract. Primary tumors of sarcoma origin are extremely rare. Herein, we present a teenager who had recurrent sarcoma of the right femur with penile metastasis.

**Case presentation:**

The 20-year-old male patient was diagnosed with sarcoma of the right femur when he was 16 days old. He was stable following combination chemotherapy with the VAC regimen (vincristine, adriamycin, and cyclophosphamide) and debulking surgery. In January 2018, five months ago, he presented with right leg pain and swelling, and a recurrent tumor was found. Following excision of the tumor showed recurrent sarcoma. However, 2 months after the operation, right thigh swelling with tenderness occurred. A firm nodule on the glans of the penis was also noted. Penile metastasis was suspected and a biopsy was performed. The final pathology report disclosed pleomorphic sarcoma with penile metastasis. Symptoms including priapism and inguinal lymph node enlargement progressed rapidly within 2 weeks. He also complained of voiding difficulty with urine retention. The patient died 35 days after admission due to pneumonia with septic shock.

**Conclusion:**

Penile metasitasis largely occurs from organs in the pelvis. To the best of our knowledge, this is the first case of a teenager with a secondary penile tumor, metastasizing from sarcoma of the bone. It presented as a palpable mass, and then progressed into priapism. The patient had a dismal prognosis and the symptoms progressed faster than his physicians anticipated.

## Background

Secondary penile tumors are a rare clinical entity, despite the rich vascularization and circulation between neighbouring organs. Fewer than 500 cases have been reported reviews since 1870 [1–3]. The vast majority of primary tumors occur in the genitourinary organs, followed by the recto-sigmoid region [4]. To date, penile tumors secondary from sarcoma origins are extremely rare [5]. Herein, we report a teenager with recurrent sarcoma with penile nodular lesions which were proven to be sarcoma metastases. We discusses the possible metastatic routes and clinical factors associations.

## Case presentation

**Fig. 1:**
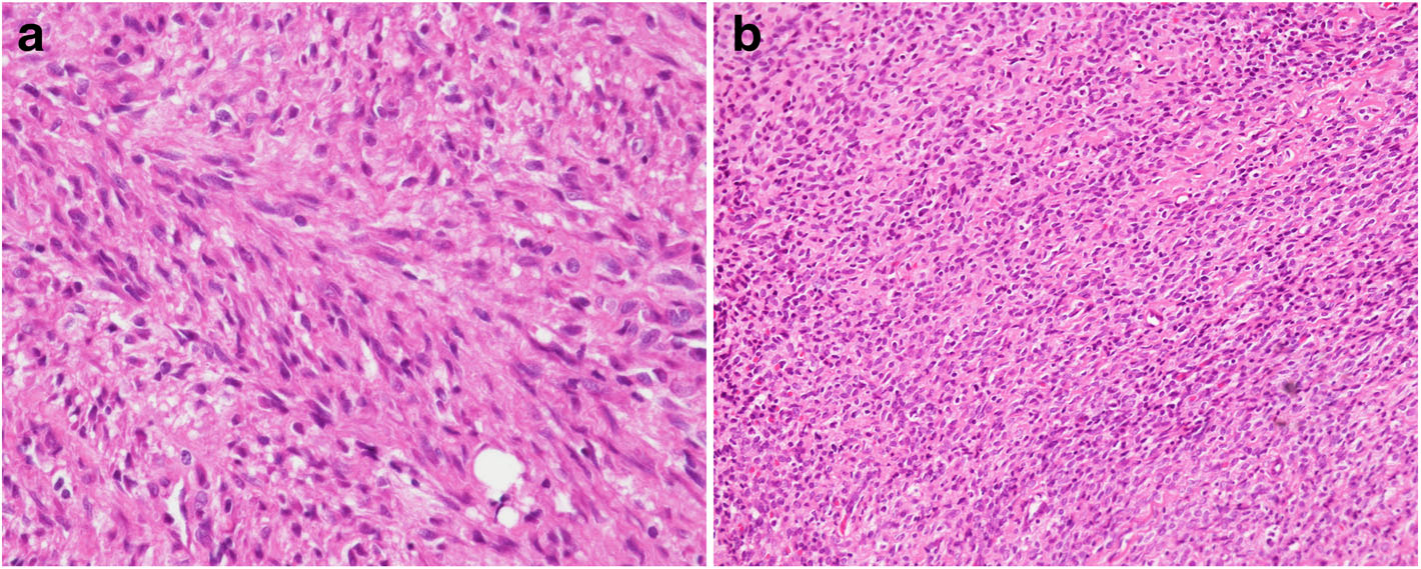
Pathology of locally recurrence tumor. **a** 400x H&E of the original low-grade neoplasm from 1999 composed of monotonous atypical spindle cells arranged in long sweeping bundles. **b** 200x H&E of a recurrent lesion in 2018 with the presence of a round cell component

The 20-year-old male patient was diagnosed with spindle cell sarcoma of the right femur in Aug. 1998, when he was 16 days old, at Mackay Memorial hospital (consent was obtained from his mother for the publication). Due to right thigh enlargement with a palpable mass, excisional biopsy were performed, and pathology disclosed a spindle cell sarcoma with hypercellularity (Fig. 1a). The patient was administered five courses of combination chemotherapy with vincristine, adriamycin, and cyclophosphamide. Debulking excision of the tumor was performed when he was 11 months old. He was regularly followed up at the orthopedics department over the next 20 years and he recieved reconstruction surgery, after which he was able to walk and run. Until 5 months ago, January 2018, he complained of progressive right thigh pain with radiation to the right lower leg, which continued for 5 months. The patient also complained of right leg numbness and weakness. Follow-up magnetic resonance imaging (MRI) revealed an infiltrative tumor mass measuring 3.6 × 3.3 × 10.4 cm in the medial proximal right thigh. The patient underwent tumor excision in May 2018. A tumor was removed from beneath the sciatic nerve and the pathology report showed recurrent sarcoma with a round cell pattern (Fig. 1b).

**Fig. 2:**
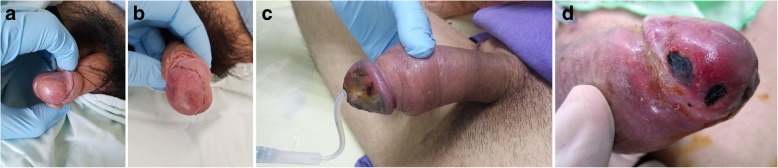
Gross pictures. **a** Painless firm nodules over the glans of the penis on admission **b** Mild swelling with purple congestion change. **c** Priapism with urine retention and an s/p nephrostomy tube and penis biopsy **d** Gangrene change over the glans of the penis

**Fig. 3:**
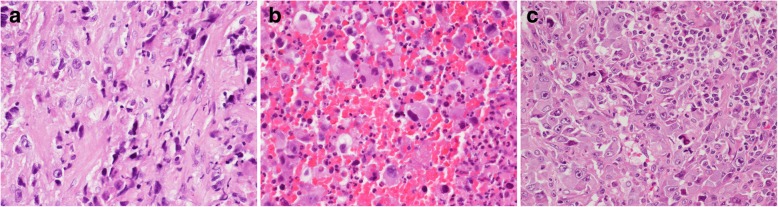
Pathology of metastatis tumors. The presence of high grade anaplastic epithelioid cells arranged in a nodular pattern. Frequent mitosis and abundant lymphoplasmacytic infiltrates can be found in these metastatic lesions. **a** Right thigh open biopsy. **b** Penile biopsy **c** Inguinal lymph node biopsy

Two months after the surgery, the patient complained of progressive right thigh swelling with tenderness and swelling of the glans penis with painful nodules. Leg computed tomography (CT) revealed a huge intraoseous cystic lesion in the right femoral shaft. Penile swelling was thought to be an balanitis at first. Urologists were then consulted for the penis lesion and 2 firm nodules about 1 to 2 cm in size were identified on the glans of the penis. No discharge, ulceration or necrosis was noted on the the overlying skin of the glans (Fig. 2a). Morning erection were unaffected; however, 3 days later the nodules had not shrunk and became a mild purple in color (Fig. 2b). Furthermore, progressive penile pain and swelling from the glans to the shaft was noted (Fig. 2c). Priapism with venous thrombosis was suspected; He compained of voiding difficulty and urine retention, but only a 14 Fr Foley tube could be inserted due to compression. In Aug. 2018, one week after admission, biopsies of the right leg and penis were performed. During the operation, a 16 Fr cystoscope could only reach the distal urethra and a 6 Fr ureteroscope revealed mucosal swelling throughout the urethra. Gross hematuria with blood clot retention in the urinary bladder and right inguinal lymph node enlargement was noted. Two weeks after admission, a suprapubic cystostomy was performed for blood clot evacuation, as it was difficult to insert a cystoscope into the urethra. An open biopsy of the right inguinal lymph node was also performed. The penile biopsy showed a hypercellular lesion with an infiltrating border, spindle fascicles with collagen deposition, and cellular pleomorphism. Histopathological examination of the recurrent tumor on the right thigh, penile lesion and inguinal lymph node showed presence of the same type of enlarged bizarre tumor cells with prominent cellular atypia. Immunohistochemistry staining of the recurrent tumor and metastasis lesion showed Gata3 (−) CD45 (−) ALK-P (−) melanA(−), S100 (−) and INI-1 with an intact expression. A FISH (Fluorescence in situ hybridization) study showed the absence of the ETV6-NTRK3 gene (a characteristic gene fusion identified in infantile fibrosarcoma). Thereby ruling out urothelial carcinoma, lymphoma, inflammatory myofibroblastic tumor, melanoma, infantile sarcoma and epithelioid tumor. The final pathology was penile metastasis and inguinal lymph node metastasis of high-grade pleomorphic/epithelioid sarcoma (Fig. 3a,b,c). Compared to previous tumor morphology (Fig. 1), the tumor recurred became of spindle cell with round cell pattern. And then, it transformed with biphasic bizarre anaplastic epithelidoid cell (Fig. 3) in 2 months.

Four weeks after admission, the patient still had persistent priapism with gross hematuria, and poor appetite with progressive cachexia. The biopsy wound over the glans developed gangrene (Fig. 2d). Fever with pneumonia occurred during the hospitalization. The patient died due to septic shock with respiratory failure 35 days after the penile swelling first ocurred.

## Discussion and conslusion

The majority of secondary metastatic penile tumors origniate from adjacent organ systems in the pelvis, including the genitourinary organs or the rectum. Previous reviews reported that the most common metastatic organs were the prostate and bladder, followed by the colorectal system. Fewer than 10% of cases are from other organs, such as the lung, liver and bone [2].

Nearly 500 cases of penile metastasis tumors have been reported, but only 3 previous cases have reported a primary tumor site with sarcoma origin. The first reported case was a 37-year-old male patient with penile metastasis from the primary site of chondrosarcoma in the jaw [6]. The second case was a 35-year-old male who had primary extraosseous penis osteosarcoma [7] and the third case was a 42-year-old male with a history of ischial tuberosity osteosarcoma with pulmonary metastasis and a painful penile metastatic lesion [5].

To the best of our knoweldge, the present study is the first case of sarcoma with multiple recurrences, where the first metastatic site was the penis. In addition, the present case is the first reported in a teenager. In a previous case series, the patients with penile metastasis were aged between 60 and 80 years [8] and most patient had disseminated disease condition. The metastasic route and mechanism have yet to be elucidated. The most accepted mechanisms of metastasis are as follows: 1) A retrograde venous route, 2) a retrograde lymphatic route, 3) arterial spread, 4) direct extension, and 5) implantation secondary to instrumentation [1] The retrograde venous route is the most likely mechanism of metastasis because there is communication between the dorsal penile venous system and the pelvic organs, and therfore bladder and prostate tumors are able to reach the penile circulation. In our case, the retrograde lymphatic route could not be ruled out, because he also presented with right leg swelling and lymph node enlargement.

It is difficult to identify penile lesions except by an open biopsy. There is no classic presentation or symptom complex in penile metastasis. Our patient had unusual symptoms of priapism, urine retention and gross hematuria. Previous cases series, mostly presented with painless nodule lesions over the penis, followed by priapism and pain [1, 2, 9]. However, the metastatic manifestation of priapism is uncommon. The mechanism could be either occlusion of the draining vein or secondary tumor thrombus occlusion of carvernosal spaces. Unlike a previous case report, urine retention and gross hematuria developed after priapism in our case. Malignant priapism was also noted in the case with disseminated metastasis; however, only locally advanced recurrence in the right thigh with primary metastasis to the penis was noted in our case. Unfortunately, the disease progressed rapidly before the biopsy report revealed the final diagnosis. Due to the rapidly-changing morphology of recurrent tumor cells, and shorter recurrent intervals, tumor progression with penile metastasis was likely.

Penile metastasis is an indicator of a poor prognosis. The median survival time after the diagnosis of penile metastasis is 10 months (range 6–18 months) and the patient presenting with priapism and those with metastasis from non-urologic tumors have a significantly worse prognosis [9]. Our 20-year-oldeee male patient died 35 days after penile metastasis due to cachexia status and septic shock. We believe that the poor disease outcome and dramatic progression were related to the tumor cell transformation. The recurrent sarcoma underwent anaplastic transformation into high grade pleomophic sarcoma, which then progressed with both local recurrence and penile and right inguinal lymph node metastasis in 2 months.

Secondary malignancy of the penis rarely occurs in young patients, and it mostly originates from the genitourinary organs. A literature review showed that the current case is the first case of metastatic penile tumor originateing from sarcoma of the femur in a teenager. He presented with a palpable mass and subsequently developed priapism and gross hematuria. Because the overall outcome is poor, most patients will need early palliative or supportive care.

## Data Availability

Records and data pertaining to this case are in the patient’s secure medical records in Mackay Memorial Hospital.

## Abbreviations

CT: Computed tomography
FISH: Fluorescence in situ hybridization)
MRI: Magnetic resonance imaging
VAC: Vincristine, adriamycin, and cyclophosphamide
